# Feasibility Analysis of LTE-Based UAS Navigation in Deep Urban Areas and DSRC Augmentation †

**DOI:** 10.3390/s19194192

**Published:** 2019-09-27

**Authors:** Euiho Kim, Yujin Shin

**Affiliations:** 1 Mechanical and System Design Engineering Dept., Hongik University, Seoul 04066, Korea; 2 Autonomous Vehicle & Intelligent Robotics Program., Hongik University, Seoul 04066, Korea; syj3558@naver.com

**Keywords:** unmanned aircraft systems, Global Navigation Satellite Systems, LTE

## Abstract

The current autonomous navigation of unmanned aircraft systems (UAS) heavily depends on Global Navigation Satellite Systems (GNSS). However, in challenging environments, such as deep urban areas, GNSS signals can be easily interrupted, so that UAS may lose navigation capability at any instant. For urban positioning and navigation, Long Term Evolution (LTE) has been considered a promising signal of opportunity due to its dense network in urban areas, and there has recently been great advancement in LTE positioning technology. However, the current LTE positioning accuracy is found to be insufficient for safe UAS navigation in deep urban areas. This paper evaluates the positioning performance of the current network of LTE base stations in a selected deep urban area and investigates the effectiveness of LTE augmentations using dedicated short range communication (DSRC) transceivers through the optimization of the ground LTE/DSRC network and cooperative positioning among UAS. The analysis results based on simulation using an urban canyon model and signal line of sight propagations show that the addition of four or five DSRC transceivers to the existing LTE base station network could provide better than 4–6 m horizontal positioning accuracy (95%) in the selected urban canyon at a position of 150 ft above the ground, while a dense LTE network alone may result in a 15–20 m horizontal positioning error. Additionally, the simulation results of cooperative positioning with inter-UAS ranging measurements in the DSRC augmented LTE network were shown to provide horizontal positioning accuracy better than 1 m in most flight space, assuming negligible time-synchronization errors in inter-UAS ranging measurements.

## 1. Introduction

The signals of Global Satellite Navigation Systems (GNSS) are unreliable indoors and in urban areas, and the development of alternative positioning methods for these areas still remains a challenging problem while the demands on the positioning technologies in GNSS-denied environments escalate in various applications including public safety assurance in disastrous situations [1,2], smart parking systems [3,4], and operations of unmanned systems, to name a few. In a relatively small area such as a parking lot or a shopping mall, Radio Frequency Identification (RFID) and Ultra Wideband (UWB) sensors have been successfully deployed to track cars or humans [5,6]. For the problem of urban navigation, various kinds of vision-based techniques have also been introduced and most of them are based on optical-flows, feature extractions, and feature matching algorithms [7,8]. Recently, Long Term Evolution (LTE)-based positioning has been considered as one of the promising signals of opportunities (SOP) due to its extensive existing network in urban areas [9,10,11,12,13]. Therefore, the LTE positioning service could provide better performance in urban than rural areas, and thus seems to appropriately complement GNSS. LTE uses orthogonal frequency division multiple access in the downlink and single carrier frequency division multiple access for the uplink access. The reference signals of LTE systems include synchronization signals, cell specific reference signals, sounding reference signals, and positioning reference signals. These signals are needed to demodulate data signals and can also be used for ranging measurement. With ranging measurements, the observed time difference of arrival method is commonly used for positioning, and commercial LTE positioning systems with ranging accuracies of 10–50 m have been introduced [14].

To enable an autonomous flight of unmanned aircraft systems (UAS) in urban outdoor areas, vision or LTE seem to be better sensors than RFID and UWB because the coverage of RFID is relatively short and the wide spectrum of UWB may become a serious interference source to other electronic systems. In fact, there has been intense research on development of a robust estimator and a vision-based autonomous flight of UAS in urban areas [15,16,17,18,19]. Chao et al. modeled the UAS navigation problem in large-scale complex urban areas as a partially observable Markov decision process and proposed a deep reinforcement learning approach to compute optimal paths and control signals [15]. Unlike the conventional Kalman filter based estimator, the authors of [16] proposed a nonlinear observer that could correct unbounded large position errors of GNSS with acceleration measured by an inertial measurement unit (IMU). Tomic et al. introduced hardware and software architectures with multiple cameras onboard toward an autonomous navigation of UAS in deep urban areas [17]. References [18,19] introduced a vision-based position estimation and control with single or multiple UAS. The advantage of those vision-based approaches is that they do not require an infrastructure, however, their sensitivity to the light conditions may become problematic during critical operations.

The recent LTE positioning technology has also been applied to UAS navigation in urban areas, and the reliability of LTE positioning would not be an issue due to its extensive network of base stations. Khalife et al. proposed a framework for precise UAS navigation using carrier phase differential cellular measurements [20,21]. Shamaei et al. presented a software defined radio design that could acquire, track, and produce range measurements from a user to LTE base stations [22]. Rufa et al. investigated the possible improvements of UAS positioning accuracy by fusing LTE ranging measurements with GNSS and vision [23]. In the near future, an LTE module is expected to be onboard most UAS because the Unmanned Aircraft System Traffic Management (UTM) operation will require UAS to send vehicle status, including position, velocity, flight trajectory, and health, to the ground infrastructure through LTE communication channels [24]. Therefore, it would be advantageous for UAS if the LTE module required for UTM communication could also serve as a back-up positioning sensor. However, LTE alone may not be able to provide sufficient positioning capability for UAS to safely fly at a low altitude of an urban canyon due to the relatively poor ranging accuracy of LTE compared to GNSS [25]. Thus, it is necessary that other sensors augment LTE to obtain better positioning accuracy of UAS in urban areas. 

For the augmentation of LTE positioning, dedicated short range communication (DSRC) is an excellent candidate, because DSRC at 5.9 GHz is also expected to be onboard to support ground-to-air and/or air-to-air UTM communications [24]. DSRC transceivers have been used as communication devices in the automobile industry, and ranging estimation using DSRC has been also investigated in past studies [26,27]. In these prior studies, DSRC ranging was obtained from radio signal strength (RSS), time of arrival (TOA), time difference of arrival (TDOA), and two-way communications. The ranging estimation using RSS is simple, but small RSS errors may result in a large ranging error. The TOA approach may provide decimeter levels of ranging accuracy but requires good time synchronization between DSRC transceivers. The TDOA approach eliminates the needs of time synchronization by using the time difference of the signal transmitted from ground DSRC transceivers, but nonetheless still requires time synchronization between the ground DSRC transceivers. Two-way communication ranging does not require any kind of time synchronization but requires frequent signal transmission. The ranging accuracy of those methods was claimed to be better than 2 m (95%) when a line of sight between two transceivers exists [27]. The coverage of a DSRC transceiver is reported to be around 1.0 km, which seems to be adequate in an urban area. 

This paper investigates the augmentation alternatives of LTE with DSRC for safe and efficient UAS navigation in an urban canyon. DSRC augmentation is considered in two different ways in this paper. First, an optimized LTE/DSRC ground network is established to enhance the LTE position solution and the network layouts are determined using binary integer programming [28]. The optimized network will only choose effective LTE base stations and add a minimal number of new DSRC transceiver sites to the existing LTE network. For the demonstration of the optimized network, Gangnam downtown in Seoul, South Korea will be used as a test site. Second, in addition to the LTE/DSRC ground network, DSRC is used to measure the inter-range between UAS to implement cooperative positioning that uses UAS as another ranging source [26]. This method could further enhance UAS position when there are other UAS flying nearby. 

This paper will first present the LTE positioning performance with the current LTE base stations in Gangnam downtown in Seoul, South Korea. The binary integer linear programing formulation for the design of optimal hybrid LTE/DSRC networks will follow. Then, the recommended optimized network and its positioning performance will be presented. After evaluating the benefits of cooperative positioning with nearby UAS, conclusions will follow.

## 2. LTE Positioning Accuracy with the Current Network in Deep Urban Areas in South Korea

**Figure 1:**
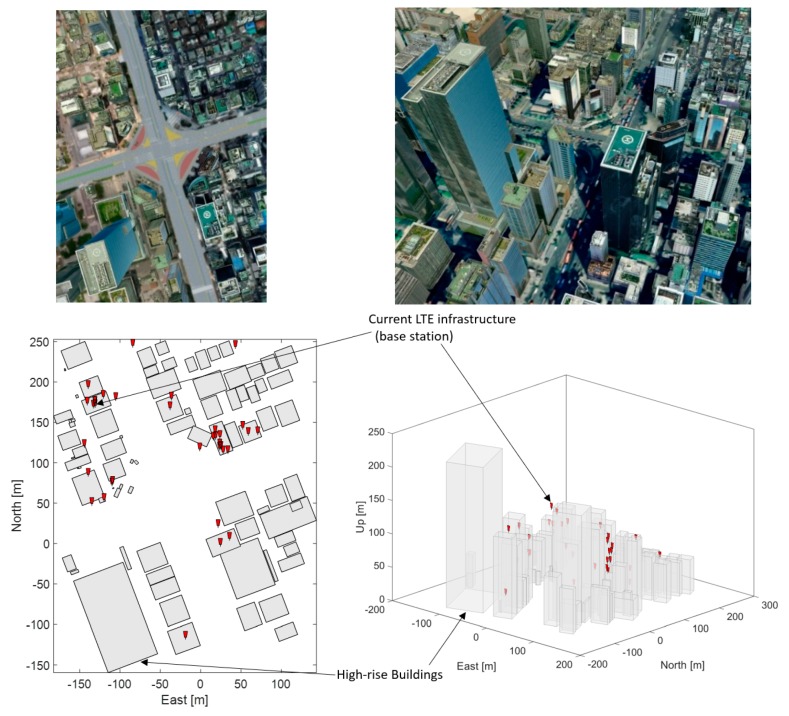
Actual image of Gangnam subway station intersection located at 396, Gangnam-daero, Gangnam-gu, Seoul, South Korea (top), and virtual city model with the current Long Term Evolution (LTE) base station network at the intersection of Gangnam subway station in Seoul, South Korea (bottom).

For the evaluation of the LTE positioning performance in deep urban areas, a virtual city model was constructed based on a digital map of Gangnam subway station intersection located at 396, Gangnam-daero, Gangnam-gu, Seoul, South Korea. In this region, there are a total of 35 LTE base stations as shown in Figure 1 [29,30]. Horizontal positioning accuracy is only evaluated in this paper because vertical guidance can be provided by a barometric altimeter. 

**Figure 2:**
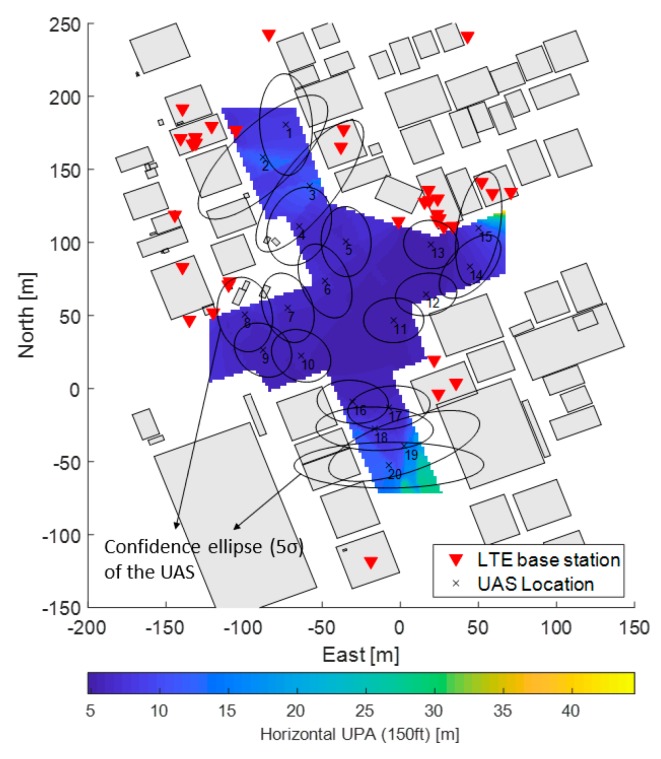
Horizontal positioning accuracy and confidence ellipses (99.999%) of 20 unmanned aircraft systems (UAS) with 35 LTE base stations.

There have been several studies for a realistic ranging accuracy of LTE and it varies depending on signal bandwidths and ranging algorithms [13,14,31]. In urban areas, in addition, multipath is a significant ranging error source that LTE ranging algorithms should be able to counteract multipath effects for a reliable position estimation. Jose et al. have recently proposed joint time-delay and channel estimation techniques that were shown to suppress multipath and provide about two times higher ranging accuracies than other algorithms [13]. Based on the research outcome, this paper assumes that the ranging accuracy of LTE is 10 m (1 sigma), and Figure 2 shows the simulated horizontal positioning accuracy of the flight space of the region of interest at a position 150 ft above ground with all of the LTE base stations shown in Figure 1. The positioning accuracy (PA) was estimated from

(1) $$P={\left(G^{T}W^{-1}G\right)}^{-1},$$

where ***G*** is a 2-dimensional geometric matrix between ranging sources and a user [9]. ***W*** is a covariance matrix of the ranging sources and is assumed to be

(2) $$W=\left[\begin{array}{cccc} \sigma_{LTE,1}^{2} & 0 & 0 & 0\\ 0 & \sigma_{LTE,2}^{2} & 0 & 0\\ 0 & 0 & \ddots & 0\\ 0 & 0 & 0 & \sigma_{LTE,n}^{2} \end{array}\right].$$

Note that Equation (1) assumes synchronized time of arrival ranging measurements for the simplicity of the analysis and LTE base stations having a line of sight to a flight space are only used at each flight space grid. In Figure 2, a 5-σ (99.999%) confidence ellipse of the positioning error is also shown around each UAS. The confidence ellipse is computed from the **P** matrix in Equation (1) and is used in this paper as a safety bound to protect from collisions with buildings and other UAS due to positioning errors. Figure 2 also shows that the positioning accuracy (1 σ) varies from 5 to 45 m, and that 12 of the confidence ellipses out of 20 UAS overlap with buildings, which indicates that the risk of collision with the surrounding buildings may be larger than 0.001% at that instant. If we consider the overlaps of confidence ellipses among UAS at the same time, the risk of collision becomes even more significant. Therefore, it can be concluded that LTE ranging accuracy of 10 m (1 sigma) alone does not provide a sufficient positioning infrastructure for UAS navigation in deep urban areas. The next subsection will investigate an optimized hybrid LTE/DSRC network layout that further improves the positioning accuracy.

## 3. Planning of DSRC Augmentation and Optimized LTE/DSRC Network

The LTE base station network has been primarily deployed for mobile communication coverage, therefore the network layout is likely to be suboptimal for positioning purposes. As an LTE base station is an extremely expensive piece of infrastructure, DSRC would be a more cost-effective augmentation candidate than an LTE base station. This paper will discuss a methodology of optimally placing additional DSRC transceivers within the existing LTE network. 

**Figure 3:**
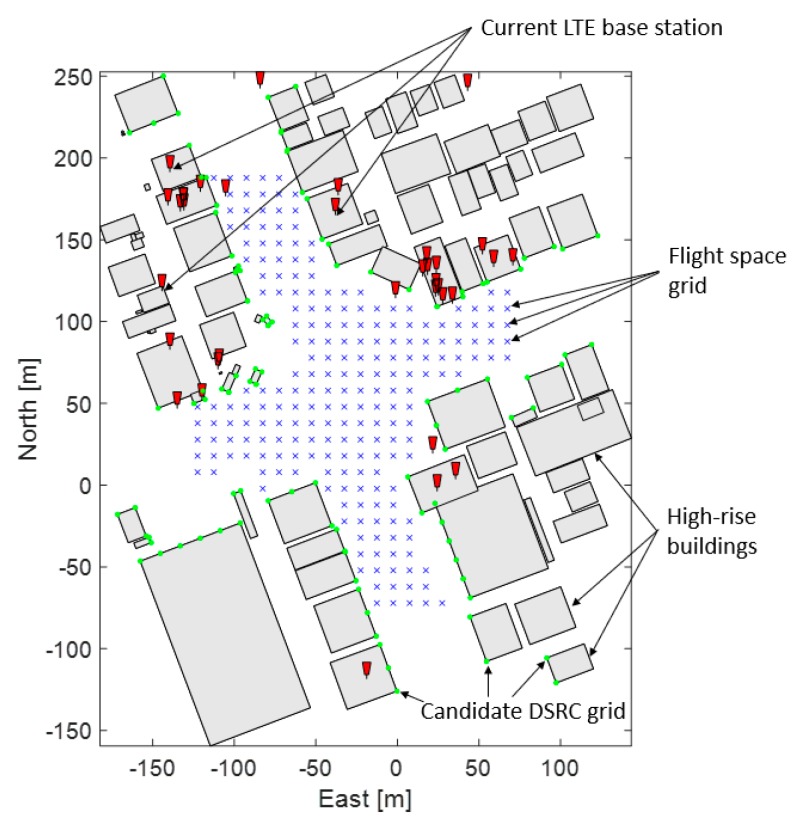
Current LTE base stations, candidate dedicated short range communication (DRSC) transceiver locations at the buildings along the main street, and flight space grids on a 2D plane in Gangnam, Seoul, South Korea.

**Figure 4:**
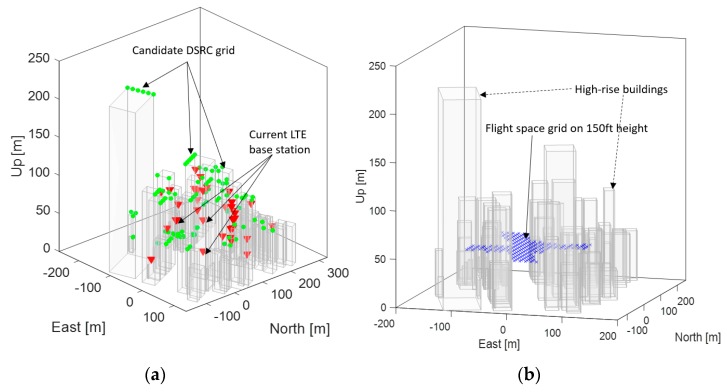
(**a**) 3D diagram of current LTE base stations and candidate DSRC transceiver locations in buildings, and (**b**) flight space at a position of 150 ft (45.72 m) above the ground, and surrounding buildings in Gangnam, Seoul, South Korea.

Figure 3 shows existing LTE base stations [29], the grids of the candidate DSRC locations, and the flight space in a 2D plane in Gangnam, Seoul, South Korea. Note that the candidate DSRC locations were placed along main streets so that UAS in the flight space grids may have a clear line of sight to the candidate DSRC locations. Figure 4a shows the existing LTE base stations and candidate DRSC transceiver locations on 3D building models. Figure 4b shows the flight space at a position of 150 ft above the ground, and surrounding buildings. For the additional DSRC placements, the ranging accuracy of DSRC was assumed to be 2 m (95%) [27], and a DSRC placement algorithm was developed by modifying the previous coverage analysis method proposed in [28]. 

The coverage analysis method in [28] searches for the optimized LTE/DSRC network by eliminating ineffective existing LTE sites and adding a minimal number of new DSRC locations. The baseline binary integer linear programming formulation is

(3) $$\begin{array}{l} \min Z=\sum_{i=1}^{n}w_{i}x_{i}=w^{T}x\\ \begin{array}{cc} \mathrm{subject}\;\mathrm{to}: & Ax\geq b\\ & Cx\leq d\\ & Fx\leq g\\ & w^{T}x\leq Z_{\min}\\ & x_{i}\in \left\{0,1\right\} \end{array} \end{array}$$

where *Z* is the weighted sum of the chosen candidate station indices and is the cost function to be minimized. **x** is a column vector consisting of (0, 1), which is the binary index of the candidate station grids of DSRC and existing LTE base stations. If a vector element of **x** is equal to 1, then the corresponding DSRC or LTE location is chosen. **w** is the weighting factor for each element of **x** and is lower for the current LTE base stations than DSRC candidate locations, which would include a larger number of existing LTE base stations. The matrix **A** is a visibility matrix that indicates line-of-sight between ranging source location grids and flight space grids. The elements of matrix **A** also consist of 0 and 1. The visibility matrix is constructed by analyzing the 3D virtual building model of the digital map of Seoul. The vector **b** is a column vector and its elements are the minimum number of ranging sources to be seen at each flight space grid. The matrix **C** is a ranging source separation matrix that prevents selection of stations located close to each other. The column vector **d** is the maximum number of chosen stations within a designated bound. The matrix **F** includes the solution sets found, and the element of the column vector **g** has an integer value less than the number of chosen candidate stations in the previous solution sets. The matrix **F** and the vector **g** drive a unique solution in each search step. *Z*_min_ is the minimum cost in a valid solution set. Equation (3) can be solved by using a linear programming solver such as MATLAB. The sensor location index of **x** at each time must provide **b** ranging sources in view at each flight space grid with no more than **d** stations inside a designated distance. During the iterative procedure, each solution **x** is unique and induces the lower *Z*_min_ at each step

**Figure 5:**
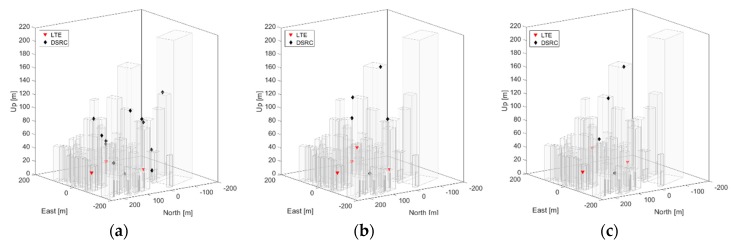
Resultant anchor locations satisfying 1, 2, and 3 m positioning accuracy (1 σ) are shown in (**a**), (**b**), and (**c**), respectively. (**a**) has a total of 15 ranging sources, (**b**) has a total of nine ranging sources, and (**c**) has seven ranging sources.

**Figure 6:**
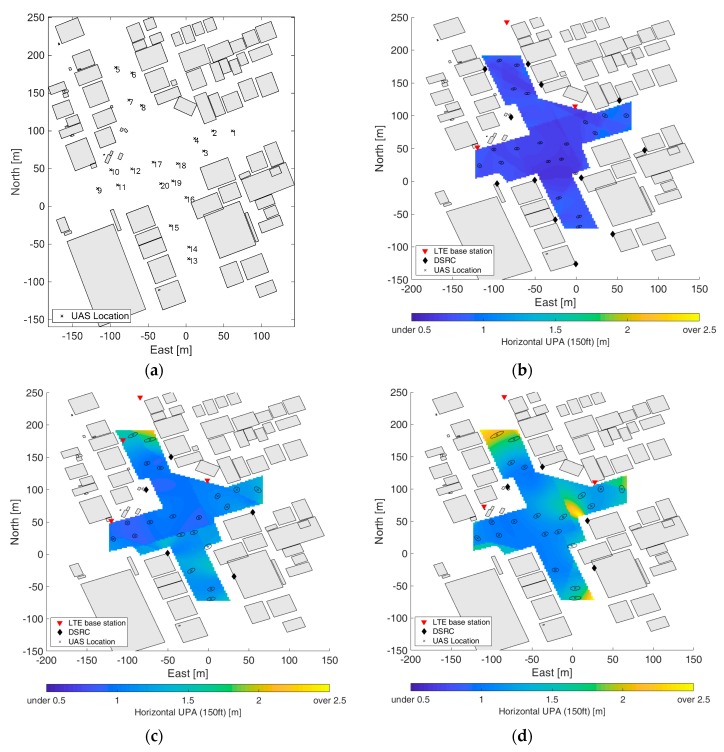
(**a**) Twenty UAS locations in the modeled environment. (**b**–**d**) Show user positioning accuracy (UPA) of the flight space and confidence ellipses of the 20 UAS in the optimized positioning network for 1, 2, and 3 m, respectively. As expected, the best user positioning accuracy is when the flight space is well surrounded by ranging sources.

The binary linear programming formulation iteratively finds valid solution sets until the required positioning accuracy is met for all of the flight space. In general, the tighter the required positioning accuracy, the more significant the resulting DRSC augmentation. Table 1 lists the characteristics of the resultant optimized hybrid positioning networks with respect to the three required positioning accuracies in terms of the number of selected current LTE base stations and additional DSRC transceivers. The layouts of the hybrid positioning networks for the three cases are shown in Figure 5, and the corresponding positioning performance and confidence ellipses of 20 UAS locations are shown in Figure 6. 

Compared to the case of the LTE base station network alone, the optimized hybrid LTE/DSRC network has a lower number of ranging sources but provides significantly better positioning accuracy and smaller confidence bounds in the entire flight space. It also seems that at least two or three UAS at the same height above the ground could fly along the same street without overlapping their confidence ellipses in the hybrid LTE/DSRC network for 3 m horizontal positioning accuracy. To allow heavier air traffic, of course, an optimized network designed to provide tighter positioning accuracy can be used, although it would require a burdensome infrastructure as already shown in Table 1 and Figure 6. However, a cost-effective approach to enhance positioning accuracy without placing extra ranging sources using infrastructure is to use cooperative positioning, which is further discussed in the next section.

**Figure 7:**
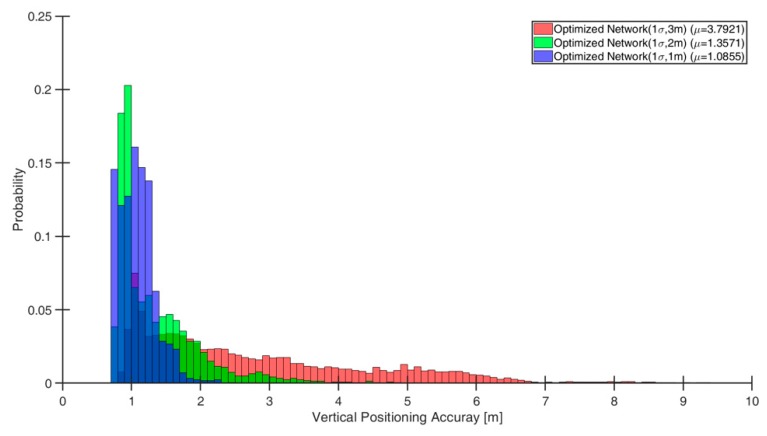
Vertical positioning accuracy distributions in the flight space using the three optimized LTE/DRSC networks.

The LTE/DSRC networks in fact have varying altitudes as shown in Figure 5, thus those networks can be utilized as a baseline set-up to see the obtainable vertical positioning performance against the number of ranging sources although the networks were not specifically optimized for vertical positioning. Figure 7 shows the vertical positioning accuracy distributions in the flight space grids for the three optimized networks. In Figure 7, the vertical positioning accuracy in many flight space grids is between 1 and 2 m (1 σ) even in the densest network with 15 ranging sources. Note that the altitude measurement uncertainty of a barometric altimeter is typically around 2 m (1 σ). Therefore, an LTE/DSRC network with 15 ranging sources would not provide a significantly better vertical positioning accuracy than a barometric altimeter does. Of course, the placement of additional ranging sources would enhance the vertical positioning accuracy but an overcrowded LTE/DSRC network may result in some other operational problems and require a large resource. A more cost-effective way of augmenting a vertical positioning with LTE/DSRC is a future research objective.

## 4. Cooperative Positioning

Inter-vehicle ranging measurements have been used to improve or maintain the estimation of relative position among vehicles during GNSS outages [32,33], which is often called cooperative positioning. In this paper, cooperative positioning uses nearby flying vehicles as ranging sources in addition to LTE or DSRC. With the additional ranging sources, UAS could further improve positioning accuracy, which will be desirable for collision avoidance when a number of UAS happen to fly close to each other or should fly in a formation. DSRC has been proposed for the cooperative positioning of autonomous cars due to its vehicle-to-vehicle ranging and communication capability, and can similarly be used for UAS cooperative positioning. For the implementation of cooperative positioning, DRSC would be used to measure inter-ranges among UAS, and to deliver UAS position solutions and the position error covariance of UAS through designated communication channels. 

When evaluating the horizontal positioning accuracy of the ground LTE/DSRC network, the coordinate error of the LTE/DSRC locations was ignored in the ranging accuracy, assuming that the coordinate is accurately surveyed. However, since the UAS position solution is used as the coordinate of the ranging source, the total ranging accuracy of the i^th^ UAS in the case of cooperative positioning can be modeled as

(4) $$\sigma_{tot,i}=\sqrt{\sigma_{DSRC}^{2}+\sigma_{Cood,i}^{2}},$$

and

(5) $$\sigma_{Cood,i}^{}\;=\sqrt{\sigma_{E,i}^{2}+\sigma_{N,i}^{2}+2\rho_{i}\sigma_{E,i}^{}\sigma_{N,i}^{}+\sigma_{U,i}^{2}},$$

where $\sigma_{Cood}^{2}$ is the variance of the ranging source coordinate uncertainty. Note that the horizontal position uncertainty of $\sigma_{E}^{2}$ and $\sigma_{N}^{2}$ in the east and north directions, respectively, is initially caused by the hybrid LTE/DSRC network. $\rho_{i}$ is the correlation coefficient between $\sigma_{E}^{}$ and $\sigma_{N}^{}$. Note that $\sigma_{E}^{}$, $\sigma_{N}^{}$, ρ can be found from using Equation (1). As stated in Section 2, the vertical uncertainty of $\sigma_{U}^{2}$ is from a barometric altimeter, which is assumed to be 2 m in this paper [34]. In this paper, $\sigma_{E}^{}$, $\sigma_{N}^{}$, $\sigma_{DSRC}^{}$ and $\sigma_{U}^{}$ are assumed to follow a Gaussian distribution. 

**Figure 8:**
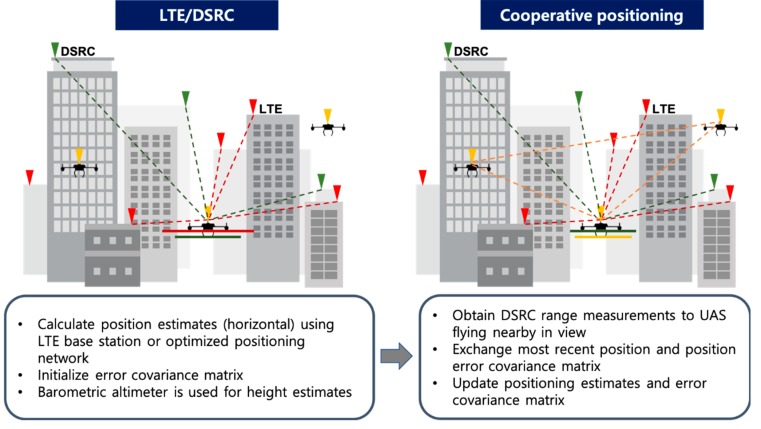
Concept of cooperative positioning operation for UAS in an urban canyon.

There are two important aspects to note in implementing cooperative positioning. First, to initiate cooperative positioning in a deep urban area, an initial position of UAS should be provided from the LTE or LTE/DSRC hybrid positioning network because the position solution of UAS is used as a ranging source coordinate as illustrated in Figure 8. The other point to note is that the DSRC inter-range measurement from each UAS would be received in an asynchronous or out-of-sequence manner. Therefore, an asynchronous Kalman filter [35] or a time compensation mechanism should be applied [36] to implement cooperative positioning among UAS in a real-time operation. However, for the baseline evaluation of the obtainable positioning accuracy using cooperative positioning, Equation (1) provides sufficient information.

**Figure 9:**
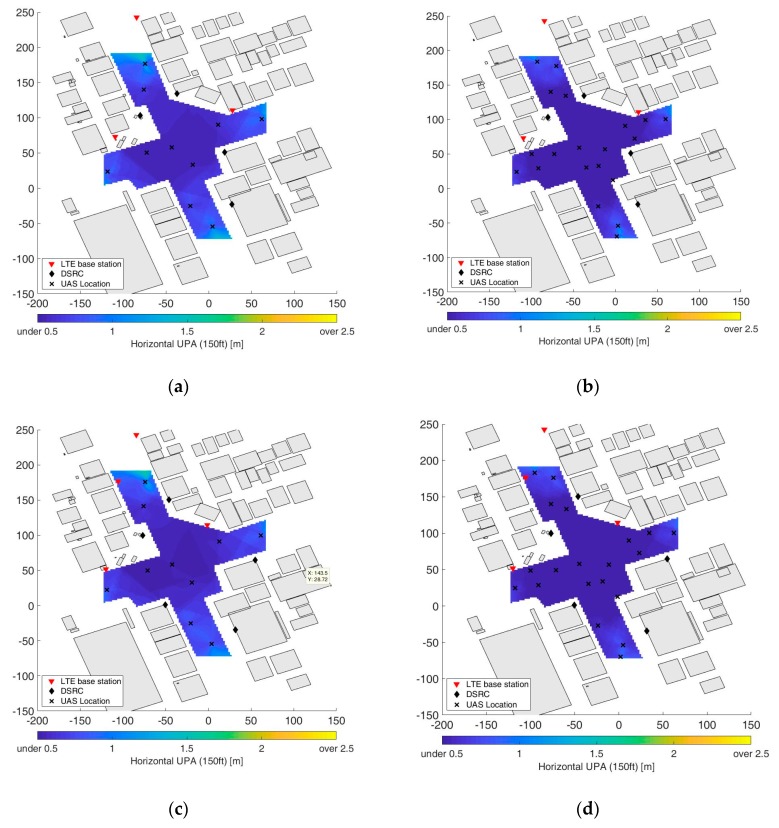

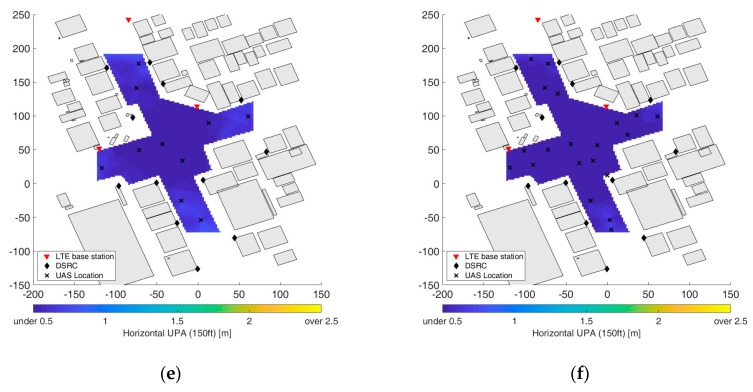
User positioning accuracy (UPA) improvement via cooperative positioning. The optimized network for 3 m (1 σ) horizontal positioning accuracy: (**a**) 10 UAS (**b**) 20 UAS; the optimized network for 2 m (1 σ) horizontal positioning accuracy: (**c**) 10 UAS (**d**) 20 UAS; the optimized network for 1 m (1 σ) horizontal positioning accuracy: (**e**) 10 UAS (**f**) 20 UAS.

**Figure 10:**
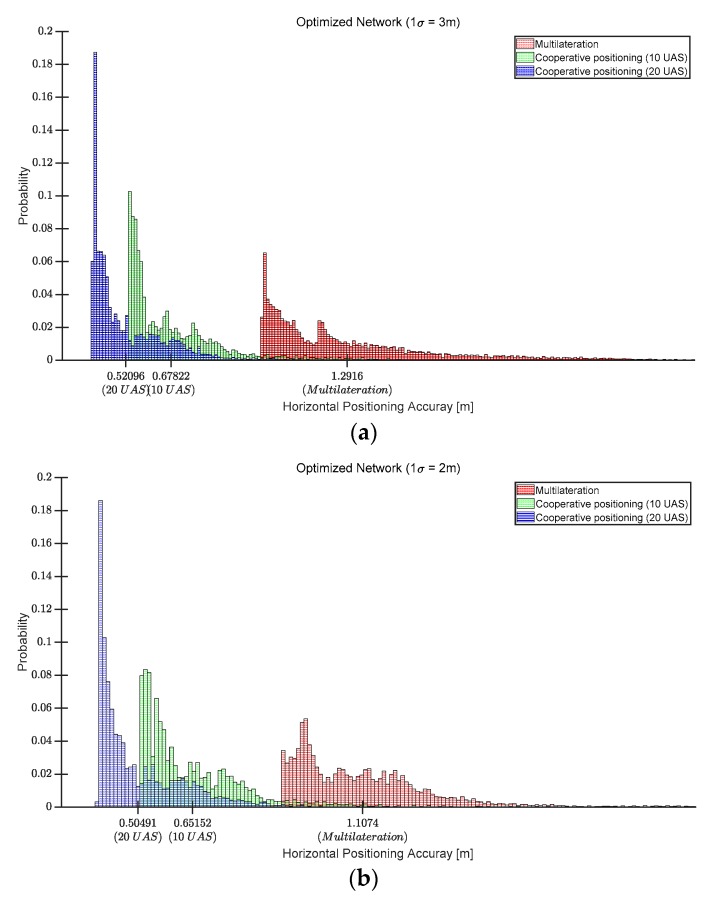

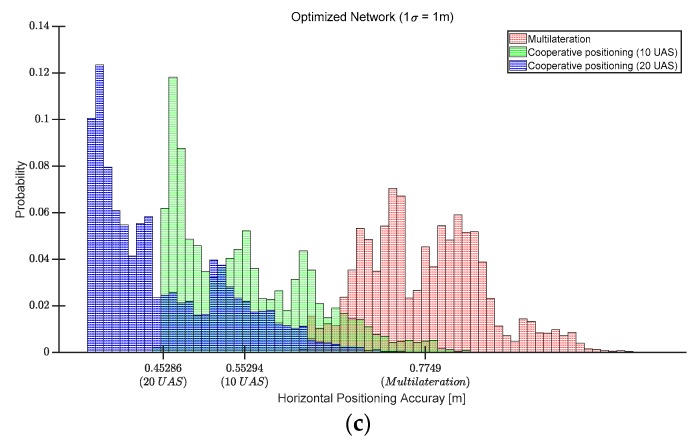
Positioning accuracy distributions of cooperative positioning with 10 and 20 UAS in the three optimized networks: (**a**) optimized network for 3 m positioning accuracy (1 σ); (**b**) optimized network for 2 m positioning accuracy (1 σ); (**c**) optimized network for 1 m positioning accuracy (1 σ).

For the evaluation of the cooperative positioning performance, 10 and 20 flying UAS were simulated in the same flight space grids. During the simulation, each flight space grid only used UAS within a line of sight as another ranging source in addition to the hybrid LTE/DSRC. Additionally, the range is measured in 3-dimensional space and projected to 2-dimensional space at an estimated aircraft height to compute a horizontal position. Figure 9 shows the cooperative positioning accuracy for 10 and 20 UAS in each optimized positioning network. Figure 10 shows the positioning accuracy distributions of the flight space for the positioning cases of the optimized LTE/DSRC network only, cooperative positioning with 10 UAS and the three optimized networks, and cooperative positioning with 20 UAS and the three optimized networks. The mean of each distribution is also indicated in Figure 10 and is better than 70 cm in the worst case. The cooperative positioning with 10 UAS and 20 UAS reduced the mean positioning error by more than 38% and 55% of the optimized networks, respectively. The histograms in Figure 10a confirm that the positioning accuracy is better than 1 m in the entire flight space except for a few grids at the edges, which would effectively provide the same positioning performance as the positioning network for 1 m positioning accuracy in Figure 5a.

From Figure 9 and Figure 10, the positioning accuracy improved as the number of aircraft increased. Using many DSRCs as ranging sources may also increase the overall packet loss rate and induce a channel access delay in the network [37,38,39]. Fortunately, today’s DSRC transceivers have more than 170 channels and apply congestion control algorithms such that about 20 DSRC transceivers have little impacts on the communication performance of the network. To implement the proposed LTE/DSRC positioning, an asynchronous Kalman filter integrated with LTE/DSRC/IMU will be appropriate, similar to a GPS/IMU integrated filter [35] because the LTE and DSRC ranging measurements will be available at UAS at different times. In [40], one cycle of a GPS/IMU integrated Kalman filter in a low cost embedded system was found to require 4300 μs at most. If an asynchronous Kalman filter sequentially processes the measurements of 23 transceivers of LTE and DSRC, a processing time of about 0.1 s will be consumed. Therefore, overall 10 Hz position update rate can be achievable with the proposed network, which should be an appropriate position update rate because most GNSS receiver output rates for UAS range from 1 to 10 Hz.

## 5. Conclusions

The analysis based on the selected urban area suggests that UAS navigation with LTE alone may not be safe due to the lack of positioning accuracy. To improve positioning performance in an urban canyon, the paper proposed an optimized hybrid LTE/DSRC network. The optimized networks having a total 15, 9, and 7 LTE/DSRC ground stations were able to satisfy the required positioning accuracy (1 σ) of 1, 2, and 3 m in the designated flight space, respectively. Cooperative positioning using DSRC was also proposed as another means of augmentation, particularly when UAS are flying closely and better positioning accuracy is desirable to avoid collision. The simulation results based on negligible time synchronization errors in inter-UAS ranging measurements showed that cooperative positioning was able to significantly enhance positioning performance. The characteristics from the simulation results suggest a cost-effective strategy of deployment of an LTE/DSRC network that could be designed to provide navigation capability and collision avoidance for relatively low-altitude air traffic in an urban canyon, in which cooperative positioning could be used to temporarily provide higher positioning accuracy in the case of increased air traffic. In the real urban environment, LTE and DSRC transceivers are also subject to multipath induced range errors. The presented UAS positioning accuracy in this paper is limited to the line of sight analysis assuming a consistent ranging accuracy in all of the flight space. In future research, multipath impacts that may be different at a user position will be investigated through further simulation and flight tests. The paper also assumes a nominal atmospheric pressure change with altitudes in urban areas, and this assumption will be verified in future studies.

## Figures and Tables

**Table 1:** The number of ranging sources of LTE and DSRC that meets the required positioning accuracy (PA).

Required Horizontal PA (1 σ)	Total Number of Ranging Sources	Number of LTE Base Stations	Number of New DSRC
1 m	15	3	12
2 m	9	4	5
3 m	7	3	4
